# The influence of the COVID-19 pandemic on the occurrence of depressive symptoms in the Croatian adult population

**DOI:** 10.1192/j.eurpsy.2024.559

**Published:** 2024-08-27

**Authors:** J. Dumic, I. Miskulin, M. Kristic, J. Kovacevic, I. Vukoja, L. Dumic, M. Miskulin

**Affiliations:** ^1^Department of Dental Medicine, Faculty of Dental Medicine and Health Osijek; ^2^Department of Public Health, Faculty of Medicine Osijek, Osijek, Croatia

## Abstract

**Introduction:**

The COVID-19 pandemic has led to a worsening of mental health among the Croatian general population. However, the overall prevalence of population depressive symptoms in Croatia over the COVID-19 pandemic is still unknown.

**Objectives:**

This study aimed to investigate the influence of the COVID-19 pandemic on the occurrence of depressive symptoms among Croatian adults.

**Methods:**

This cross-sectional questionnaire study was conducted from mid-February to mid-May 2022 period. A validated, anonymous questionnaire that contained questions regarding demographic data, as well as the Zung Self-Rating Depression Scale was self-administered to a convenient sample of adults from the city of Osijek in eastern Croatia.

**Results:**

The study sample included 500 subjects with a median age of 34 years (interquartile range 26-53), 42.4% males, and 57.6% females. According to the Zung Self-Rating Depression Scale, there were 16.2% of subjects with mild or moderate depressive symptoms. Depressive symptoms were more frequent among older subjects (61 years or older) (p=0.001), among subjects with a lower level of education (subjects with or without elementary school) (p<0.001), among subjects who were retired (p=0.005), among subjects who considered their socioeconomic status as under average (p<0.001), and among subjects who experienced death of close family member caused by COVID-19 infection (p=0.004).

**Conclusions:**

The COVID-19 pandemic increased the prevalence of depression in the Croatian general population where some sociodemographic characteristics of study subjects seem to put those subjects at greater risk considering the occurrence of depressive symptoms. Development of appropriate supportive programs that enhance the mental health of the Croatian general population during pandemics is needed to potentially prevent the occurrence of depressive symptoms and to help the general population successfully overcome this important mental health challenge.

**Disclosure of Interest:**

None Declared

